# Inter-Organizational Coordination to Improve Patient Outcomes in Multimorbid Older Patients Following Hospital Discharge – a Systematic Review

**DOI:** 10.5334/ijic.9018

**Published:** 2025-05-12

**Authors:** Wilhelm Linder, Richard Ssegonja, Inna Feldman, Robert Sarkadi Kristiansson, Jamile Marchi, Ulrika Winblad

**Affiliations:** 1 Department of Public Health and Caring Sciences, Health Services Research, Uppsala University, Sweden; 2 Department of Public Health and Caring Sciences, Child Health and Parenting, Uppsala University, Sweden; 3 Department of Medical Sciences, Respiratory, Allergy and Sleep medicine research, Uppsala University, Sweden

**Keywords:** integrated care, older patients, hospital discharge, inter-organizational collaboration

## Abstract

**Introduction:**

Health and social care systems are constantly undergoing major reforms to meet the rising demands of an increasing proportion of older patients, with many such reforms aiming to improve integration and coordination. The aim of this systematic review was to synthesize the evidence on inter-organizational coordination interventions between hospitals and outpatient (health- and social care) providers for older patients with complex needs during- and after hospital discharge.

**Methods:**

A systematic search of four databases was performed to identify interventions of inter-organizational coordination at hospital discharge for older patients with complex needs. The retrieved literature was analyzed using a narrative synthesis.

**Results:**

Twelve studies were included (seven randomized controlled trials and five non-randomized intervention studies). The most common intervention components were; needs assessments, dedicated care coordinators and multi-professional teams. Findings show that inter-organizational coordination could decrease- or even increase readmission rates, with similar findings for hospital length of stay and mortality. Furthermore, inter-organizational coordination seemed to have a positive impact on quality of life and activities of daily living.

**Conclusion:**

Inter-organizational coordination could potentially reduce health-care utilization and improve quality of life for older patients with complex needs. However, the findings remain uncertain and further research is warranted.

## Introduction

The population aged 65 and over often has complex needs that require services from a multitude of health- and social care providers, leading to substantial costs for health care systems [1]. In the OECD, the proportion of the population aged 65 and over is projected to increase from 17.3% in 2019 to 26.7% in 2050 (4.6% to 9.8% for population aged 80 and over), resulting in a growing demand for health- and social care services [2,3].

For these patient groups, health- and social care may be spread across multiple providers, often referred to as fragmented care [4]. Given the varying needs of patients with complex needs, some degree of fragmentation may be unavoidable. However, insufficient coordination and integration can lead to negative consequences for both patients (e.g. medical errors, unnecessary visits and missed care) and health care systems (e.g. inefficiency due to avoidable hospitalizations and duplication of efforts) [4]. In an effort to improve health outcomes, countries are reforming their health- and social care systems by emphasizing integration and coordination of care. In theory, this approach could offer health care providers with access to additional expertise and skills and improve the management of interdependencies [5,6]. However, collaborative efforts also tend to introduce coordination challenges, conflicting goals between health and social care service providers and questions about funding [6,7]. Currently, there is no consensus regarding how to best integrate health- and social care which hinders implementing integrated care into practice [8,9].

### Older patients with multimorbidity during hospital to home transitions

The complex needs of older patients often includes a combination of multimorbidity, such as multiple chronic diseases, physical and/or mental impairment and inadequate ability of self-care [10,11]. These health-related vulnerabilities result in a state described as frailty, where minor stressors can cause disproportionate increases in adverse outcomes [12,13]. The prevalence of frailty depends on the definition, but is reported to be from 4% to 59% among community-dwelling individuals older than 65 years of age [14]. The concept of frailty includes symptoms such as generalized weakness, poor balance, exhaustion and cognitive impairment, and clinical frailty scales are often used to determine patients care needs in hospitals [13]. This group of patients is particularly vulnerable during the transition from hospital-to-home, where the responsibility of care shifts from hospitals to outpatient health and non-healthcare providers [15].

In particular, older patients with complex needs are often referred to primary care services which in comparison to specialized care give simultaneous attention to the range of diseases affecting the patient as well addressing the patients’ functional ability [16,17]. Several studies have also shown that medication reconciliation by pharmacists and interventions by occupational- and/or physiotherapists are important post-discharge [18,19]. Furthermore, home care and other non-health care services have vital roles in ensuring the patients quality of life (QoL) and capability of performing activities of daily living (ADL) through e.g. home adaptation, transportation services, personal hygiene and meal delivery [6,20].

Studies from different countries show that care coordination, herein defined as arrangement of roles, responsibilities and tasks between different health- and social care services, is often insufficient after hospital discharge [21,22,23,24,25]. For older patients, inadequate care coordination often leads to missed or delayed care and medical errors, factors that are frequently cited as causes of avoidable readmissions and prolonged length of hospital stays (LoS) [26,27]. From a health systems perspective, improving post-discharge coordination is seen as crucial for enhancing overall system efficiency [6]. However, research at the system level indicates that coordination is challenging, often attributed to communication breakdown, resource shortages, unclear delegation of responsibility, and professional and cultural barriers [28,29,30,31].

### Coordinated care for older patients with multimorbidity

Coordination between health and non-health care organizations can be achieved at different levels, for instance at the systemic level where integration is sometimes accomplished by merging health- and non-health care providers into a single entity [32]. Such formal integration of service providers have been suggested to improve patient outcomes and system efficiency through i.e. centralizing management, facilitating communication (e.g. through harmonizing journal systems and facilitating sharing of information) and by aligning incentives and priorities (e.g. through goals and reimbursement) [32,33,34,35,36]. However, formal integration of multiple service providers is practically unfeasible in countries where health- and social care is provided by distinct and autonomous organizations. In for instance Sweden, health- and social care is organized by autonomous agencies (i.e. regions and municipalities) that are governed by different legislation and have separate commissioning and funding structures. In such non-hierarchical contexts where no single centralized actor is ‘calling the shots’, coordination between providers is primarily achieved by voluntary agreements or partnerships at the organizational and clinical (service delivery) levels, hereon referred to as *inter-organizational coordination, IOC* [37]. In practice, important aspects of integration and coordination, such as alignment of reimbursement systems and harmonization of electronic health records, can be difficult to achieve at the organizational level. Instead, coordination efforts are often focused on the service level, for example through multi- or inter-professional teams, facilitation of communication (e.g. notification systems), establishing structures for shared decision making (e.g. care conferences) and addressing cultural barriers among providers [36].

Interventions of integrated and coordinated care for older patients during hospital discharge are often interventions of high complexity. Such interventions include multiple components simultaneously and the individual components are often assumed to have synergistic effects when the intervention is tailored to specific study populations and local contexts [38,39,40,41,42,43,44,45] Commonly reported components of these interventions include geriatric risk assessments, multidisciplinary teams, and designated care coordinators [42,46].

Furthermore, many studies incorporate program theory that outlines the goals and proposed mechanisms for why a specific intervention is expected to be effective, both regarding health-related outcomes as well as system efficiency, for instance through shifting inpatient to outpatient care. [38,47,48].

In terms of effects, previous umbrella reviews report that integrated care can potentially reduce all cause readmissions by 10–30%, decrease LoS by 1–7 days and improve quality of life (QoL) [46,49]. However, positive effects are more commonly observed in disease-specific interventions, such as heart failure, which may not be adequately reflected in the needs of older patients with multimorbidity or be practically feasible in several health care systems [50].

In addition, interventions rarely show unequivocal effects [5,6,46,51], which is likely related to heterogeneity entailed by diverse intervention components [40], study populations [46], intervention fidelity [40], intervention level (micro-, meso-, macro-), context (health care system) [6,38,40], and clinical setting (e.g. preventing hospitalization or readmissions) [39,46,52,53]. As a consequence, identifying consistently effective interventions, or configurations of effective components, has been described as challenging [54,55].

#### Study rationale and aim

Previous studies of coordinated care for older patients have reported promising results regarding health-related outcomes and system efficiency. However, knowledge regarding the effects of coordinated care interventions in specific clinical settings and contexts remain elusive. In particular, hospital discharge of older patients in contexts where health- and non-health care providers, such as social care are separated into autonomous and distinct organizations are reported to be associated with higher risks of experiencing gaps in care and readmissions after hospital discharge [56]. As such, a targeted systematic review focusing on the context of inter-organizational coordination could provide valuable insights for future improvements in care coordination connected to hospital discharge.

The aim of this systematic review is therefore to investigate inter-organizational coordination (IOC) interventions for older patients with multimorbidity during hospital to home transitions. The work sought to address the following objectives:

1. To synthesize the evidence on inter-organizational coordination interventions and their individual components
2. To assess the effects of inter-organizational coordination on health care utilization and health-related outcomes.

## Methods

The systematic review was registered in PROSPERO (CRD42021226228). Modifications to the original registration in PROSPERO can be found in supplementary material A.

### Search strategy

A systematic search for English language publications published between 1^st^ of January 2010 and 22^nd^ of December 2023 was performed in Cinahl, PubMed, Scopus and Web of Science based on PRESS-guidelines [57]. The search was restricted to 2010–2023 based on a bibliometric analysis of integrated- and coordinated care that showed a large increase in publications starting in 2010. Search terms were developed together with an information specialist at Uppsala University library based on the PICO framework with the addition of context [58,59]. The accuracy of the literature search was validated by verifying that key papers previously known to the authors were found [60,61]. Additional records were obtained from reference lists of included studies and systematic reviews identified in the systematic search. An outline of the search strategy is presented in the supplementary material A (*Supplementary table 1*).

### Eligibility Criteria

Studies investigating Inter-organizational coordination for patients aged 65 and older with multimorbidity during the transition from hospital to ordinary care or nursing home care were identified. IOC was defined as interventions where a hospital and at least one non-hospital service provider both actively participated in IOC and coordinated their activities in order to achieve health goals. Coordination was broadly defined as “*the arranging of roles and tasks into an organized whole”* [21]. Furthermore, the coordinated activities had to include both health- and non-health care services, e.g. social care, home care and home adaptation services [8,21]. Hospital length of stay (LoS) and readmissions were the primary outcomes and in the final sample, additional outcomes relevant to the aims of coordinated care, e.g. health- care utilization, patient health and patient experiences, were included and analyzed. A summary of the eligibility criteria is presented in Table 1.

**Table 1:** Study inclusion and exclusion criteria.


***Population***	*Inclusion*: hospitalized patients, >65 years old, multimorbidity, complex needs (including non-healthcare needs).

***Intervention***	*Inclusion:* Inter-organizational coordination (IOC). *Exclusion*: Interventions tailored for specific diseases and interventions where hospital and non-hospital actors were not both actively participating (e.g. hospital-based care coordinators limited to referring patients to non-hospital service providers).

***Comparator***	*Inclusion:* standard care.

***Outcomes***	*Inclusion:* Length of hospital stay (LoS) and/or hospital readmissions.

***Context***	At least two distinct service providers jointly responsible for in- and outpatient health- and non-health care.

***Type of study***	*Inclusion*: Randomized Controlled Trials (RCT), Non-Randomized Studies of Interventions (NRSI) *Exclusion*: Studies without control group, single center studies (Controlled Before After, Difference-in-Difference), less than three measurements before/after intervention (Interrupted Time Series).


### Study selection

After removing duplicates, the remaining records were screened for eligibility by three researchers working independently (WL, RS, and JM). Records were considered for full text review if two of the assessors agreed that the record could not be excluded based on the abstract. In the full text review, studies were randomly assigned to four authors working independently (WL, RS, JM and IF) with each study undergoing full text-review from two authors before being included or excluded. Disagreements regarding inclusion were resolved by discussion between the two reviewing authors and a third researcher (UW). Measures of agreement (Cohen’s Kappa) were calculated for abstract screening and full text review separately [62]. In a few instances, authors of potentially eligible articles were contacted to confirm the context of at least two distinct organizations. Screening and study selection were performed using software Covidence.

### Data extraction, risk of bias assessment and data synthesis

Data from the included studies were extracted by three authors (WL, JM, and IF) using a template from the Cochrane Public Health Group Data Extraction and Assessment Template. Reported outcomes were extracted based on adjusted effects and standardized when possible [63].

Studies meeting eligibility criteria were assessed for risk of bias by three authors (WL, RS and IF). Risk of bias assessments included randomization, allocation concealment, similar baseline outcome measurements, similar baseline characteristics, incomplete outcome data, prevention of knowledge of allocation during recruitment, protection against contamination, selective outcome reporting and other biases [64]. The Risk of Bias assessment template was applied together by the three authors to two studies. The remaining studies were divided between the authors with each study being assessed by two authors working independently. Disagreements were mainly related to the domain protection against contamination and were resolved through discussion between the two responsible authors.

Qualitative evidence regarding interventions and quantitative evidence regarding effects were analyzed using a narrative synthesis. [65]. No meta-analysis was planned as the studies were expected to be interventions of high complexity [45].

## Results

### Results of the literature search

**Figure 1:**
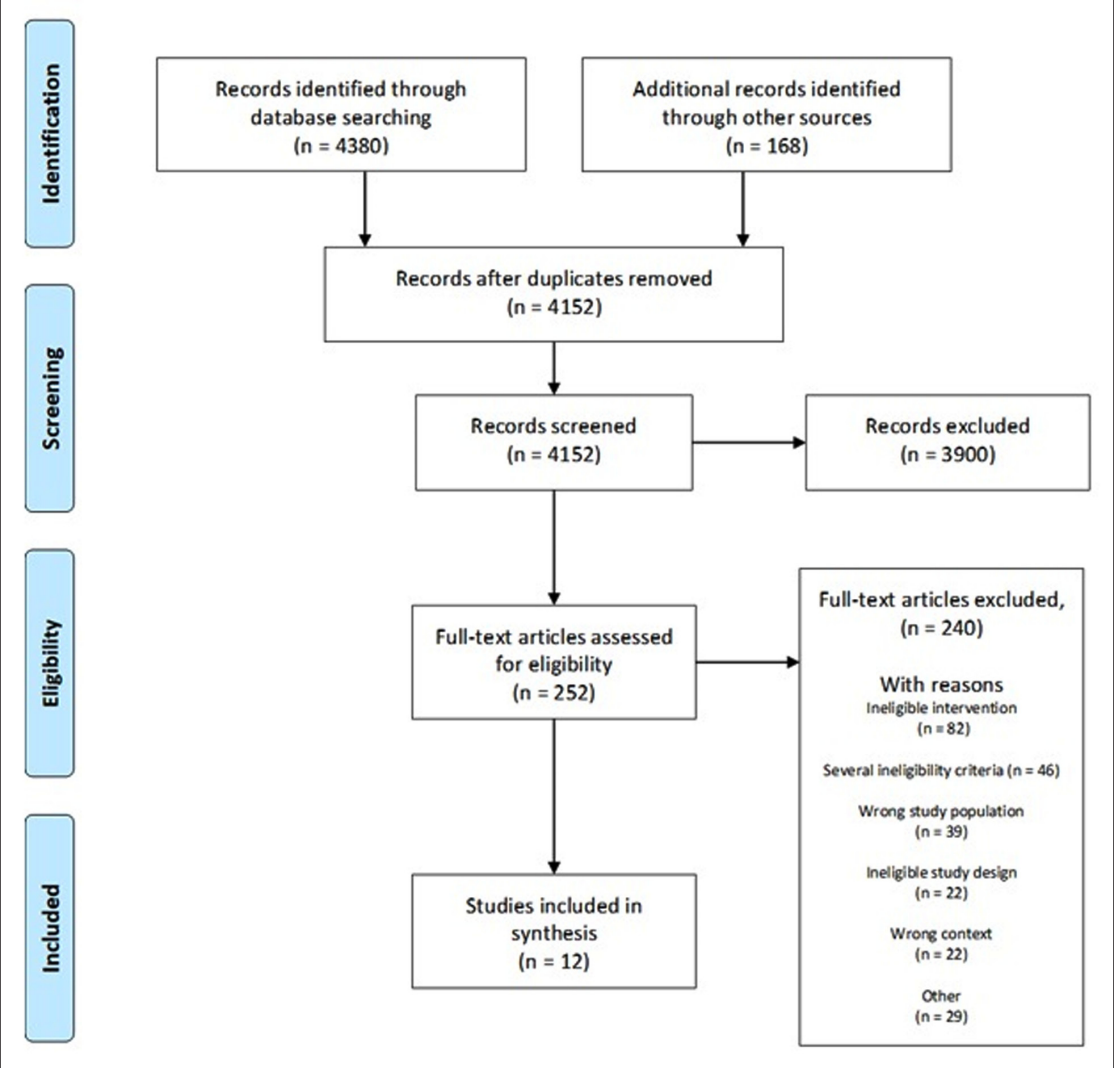
Study selection flow chart.

A flowchart of the study selection process is presented in Figure 1. The literature search yielded 4380 records. After adding 168 additional records and removing duplicates, 4152 records were screened for eligibility with 252 records identified as potentially eligible (Cohen’s Kappa ranging between 0.43–0.62). In total, 12 studies met the eligibility criteria and underwent data extraction and risk of bias assessments (Cohens Kappa ranging between 0.21 and 0.26). A brief summary of excluded articles is presented in supplementary material A.

### Description of the studies identified

#### Study characteristics

The characteristics of the 12 studies included in the review are summarized in Table 2. Seven studies were RCTs, with one being a Cluster-RCT (C-RCT) [61,66,67,68,69,70,71]. Five studies were NRSI of which four used matched control group design and one used a difference-in-difference (DiD) design [60,72,73,74,75]. All studies assessed readmissions (up to 180 days after discharge), four studies did not report LoS [61,70,72,73]. In addition to the primary outcomes, studies also assessed mortality [60,61,66,68,71], health related quality of life (QoL) [68,69,70,71], ADL [66,68,69], ED-visits [60,61,67,72,74,75], discharge destination [61,66,68,71,75], outpatient visits [60,61,68,75] and satisfaction with care [70].

**Table 2:** Characteristics of included studies.


AUTHOR, YEAR	COUNTRY	TYPE OF STUDY	PARTICIPANTS (n, TOTAL)	INTERVENTION AND KEY CHARACTERISTICS	DURATION OF FOLLOW-UP AFTER DISCHARGE (WEEKS)	OUTCOME ASSESSMENT (DAYS)	PARTICIPATING SERVICE PROVIDERS	REPORTED OUTCOMES

Berntsen 2019	Norway	NRSI: Synthetic RCT,	1218	**Patient Centered Team (PACT)** Cross-organizational multidisciplinary geriatric team.	As needed.	30–180	Hospital and municipality.	Readmission, LoS, Mortality, Emergency- and planned outpatient visits

Buurman 2016	Netherlands	RCT	674	**Transitional Care Bridge Program** Comprehensive Geriatric Assessment, multidisciplinary team	24	30–180	Hospitals and affiliated home care organizations.	Readmission, Mortality, ADL, Discharge destination

Cordato 2018	Australia	RCT	43	**Regular Early Assessment Post-Discharge (REAP)** Conjoint visits by geriatrician and nurse to nursing homes	4	180	Hospitals and nursing homes.	Readmission, LoS, ED-visits

Crilly 2011	Australia	NRSI: Quasi-Experimental	177	**Hospital in the Nursing Home (HINH) Admission avoidance program** Outreach service from hospital to nursing homes.	As needed.	28–365	Hospital and nursing homes.	Readmission, LoS, ED-visits

Jenq 2016	US	NRIS: DiD, ITS	<30 000	**Greater New Haven Coalition for Safe Transitions and Readmission Reductions program (Co-STARR)** Connecting with community services.	4	30	Hospitals and community partner organization.	Readmission

Meyer 2022	Germany	RCT	110	**Tailored Intersectoral Discharge Program (TIDP)** Cross-organizational multidisciplinary geriatric team	As needed.	30–180	Hospital and primary care providers	Readmission, LoS, Mortality, QoL, Discharge destination, Self confidence

Robert 2021	Canada	NRSI: DiD, ITS	1926	**Sub-Acute Care for Frail Elderly (SAFE)** Restorative care in a long-term care home.	4	30	Hospital and long-term care organization.	Readmission, LoS, Emergency- and planned outpatient visits, Discharge destination

Rosstad 2017	Norway	Cluster-RCT	304	**Patient Trajectory for Home-dwelling elders (PaTH)** Improved procedures for communication and follow-up.	4	30–365	Hospital and municipalities.	Readmission, LoS, Mortality, ADL, QoL, Outpatient services, Discharge destination

Sahota 2017	UK	RCT	212	**The Community In-reach Rehabilitation and Care Transition** Co-localization in hospitals and rehabilitation.	As needed.	28–91	Hospitals and community partner organization.	Readmission, LoS, ADL, QoL

Sorensen 2021	US	NRSI: Retrospective cohort study	2964	**Collaboration between Primary Care-based Clinical Pharmacists and Community-based Health Coaches**. Patient empowerment and medication management.	4	30–90	Hospitals and community-based organization.	Readmission, ED-visits

Thygesen 2015	Denmark	RCT	531	**Municipality based post-discharge follow-up visits**. Conjoint visits by primary care physicians and municipal nurses.	8	30–180	Hospitals and municipalities.	Readmission, Mortality, Emergency- and planned outpatient visits, Discharge destination

Wong 2011	Hong Kong	RCT	555	**Health-Social Partnership Transitional Care Management Program (HSTCMP)**. Conjoint visits by nurses and social care services.	4	28–84	Hospitals and social service centers.	Readmission, QoL, Self- Efficacy, Satisfaction with care


##### Study population

For RCTs, sample sizes ranged from 43 to 678 and for NRSIs the sample size ranged from 177 to 30 568. The mean age in the intervention groups ranged between 77–84 years (77–86.5 in control groups) and 59–76% (29–75% in control groups) were female. Five studies exclusively enrolled community- dwelling patients [66,68,69,70,72], two studies exclusively enrolled nursing home residents [67,74] and five studies had no inclusion criteria for patient living arrangements before hospitalization [60,61,71,73,75].

##### Risk of bias

In addition to the lack of randomization, three NRSIs had high or unclear risk of bias in relation to allocation concealment [60,74,75]. High risk of bias was also found for the dimensions similarity in baseline characteristics [73,74,75] and protection against contamination [61,66]. Unclear risk of bias were mainly found for similarity in baseline outcome measurements [60,67,70,74,75] and protection against contamination [67,69,71,72,75]. A summary of the risk of bias assessments is presented in Supplementary material A (*Supplementary figures 2 and 3)*.

##### Context

The studies were performed in the US [72,73], Norway [60,68], Australia [67,74], Canada [75], Denmark [61], Germany [71], the Netherlands [66], Hong Kong [70] and in the UK [69]. IOC was reported both at the organizational level between e.g. regions (hospitals) and municipalities [60,61,68] or other community based organizations [66,69,73] as well as at the level of service delivery, e.g. between hospitals primary care providers [71], nursing homes/long-term care facilities [67,74,75], home care and social care providers [70]. Commonly reported professions were primary care physicians [67,69], physio- and occupational therapists, social workers, pharmacists and nurses [61,72]. Furthermore, five studies reported the utilization of physicians and nurses with geriatric expertise [66,67,71,74,75].

### Intervention components

Seven studies reported descriptions of program theory and included theoretical reasoning as to why the individual components of the interventions would be effective [60,66,67,70,72,73,75]. Across studies, interventional components were divided into non-clinical, pre-discharge, transitional and post-discharge components, see Table 3. The interventions, components and their effects on primary outcomes are presented in Supplementary material B where it is possible for readers to filter based on specific components.

**Table 3:** Components and effects of interventions. Components that were found more than once are presented here together with effects on three outcomes (readmissions, LoS and mortality). For a more detailed description (including involved professions), see supplementary material B. Arrows indicate the direction of effect. Effect within 0.95–1.05 are presented as —. Follow-ups: H = Home, R = Remote, Blank = not reported.


AUTHOR	INTERVENTION TARGETED TO NH-RESIDENTS	NON-CLINICAL COMPONENTS		PRE-DISCHARGE COMPONENTS			TRANSITIONAL COMPONENTS			POST-DISCHARGE COMPONENTS					READMISSIONS	LENGTH OF STAY	MORTALITY
			
		PARTNERSHIPS OR AGREEMENTS	WORKFLOW IMPROVEMENT	SHARED PATIENT ENROLLMENT	NEEDS ASSESSMENT (FUNCTIONAL, SOCIAL, COGNITIVE)	CARE PLAN	PERSONAL/ACTIVE HAND-OVER	DEDICATED COORDINATOR	HOSPITAL VISIT BY OUT. PAT. CARE	MULTI-PROFESSIONAL FOLLOW UP	FOLLOW-UP (HOME/PHONE) VISITS	INTERMEDIATE CARE UNIT	POST DISCHARGE PLAN	RECONCILIATION MEETINGS			

Berntsen 2019		X	X	X		X		X		X	H, P		X	X	↓	↓	↓*

Buurman 2016					X	X	X	X	X		H				↑		↓

Cordato 2018	X									X	H				↓*	↓*	

Crilly 2011	X			X				X			H			X	—	↓*	

Jenq 2016		X	X		X			X			P	X		X	—		

Meyer 2022					X		X			X				X	—	↑	↑

Robert 2021	X	X	X	X	X			X		X		X			↑	↓*	

Rosstad 2017			X		X		R			X	H		X	X	↓	↓	↓

Sahota 2017					X	X			X	X	H				↑	↓	

Sorensen 2021		X				X	X			X	H		X		↓*		

Thygesen 2015							R			X	H		X		—	↑	↓↑

Wong 2011		X	X		X	X		X			H, P			X	↓*		


#### Non-clinical components of IOC

Six of the twelve studies reported non-clinical intervention components. These components were not directly aimed at e.g. providing diagnosis, treatment or care of patients yet still considered important for IOC. Five studies reported formal agreements or partnerships that included division of responsibilities and/or allocation of financial-and human resources between the service providers [60,70,72,73,75]. Additionally, five studies reported cross-organizational structures and activities aimed at improving service delivery and workflows, e.g. cross-organizational boards and meetings to create conditions that would improve adherence to study protocols [60,68,70,73,75].

#### Clinical components of IOC

##### Pre-discharge components

Intervention components during the initial hospital visit included assessments of patients’ functional, social and cognitive needs [66,68,69,70,71,73,75], care planning [60,66,69,70,72] and fast track transitions from the emergency department to post-discharge care [74]. Most studies enrolled patients through targeted screening for patients of high risk for readmission and/or functional decline (both medical and non-medical criteria) and in three studies outpatient service providers were actively involved in enrollment, i.e. shared decision-making [60,74,75].

##### Transitional Components

Several studies included components specifically aimed at facilitating the transfer of patients to their homes. These included hand-over meetings between hospitals and outpatient service providers, either conducted at the hospital or via remote sessions (e.g. telephone or digital meetings) [61,66,68,71,72], the designation of care coordinators [60,66,70,73,74,75], and hospital visits by outpatient service providers [66,69].

##### Post-discharge components

During the post-discharge phase, all studies reported follow-up visits at home or remotely [60,70,73]. Eight out of the twelve studies organized follow-up care using multi-professional teams [60,61,67,68,69,71,72,75]. Other components across studies were referrals to community services [69,73,75], intermediate care units [73,75], post-discharge care plans [60,61,68,72], and patient status meetings between the professionals involved in the follow-up [60,68,70,71,74].

##### Components of standard care

In addition to medical treatment for the acute disease, standard of care consisted of pre-discharge components such as in-hospital rehabilitation and geriatric risk assessments [66,71,76]. Transitional components included referrals to medical specialists and community services [60,70,76], and information sharing to outpatient services through discharge letters or electronic communication [60,61,73]. Only two studies reported follow-up activities in standard of care [67,73].

### Intervention effects

**Figure 2:**
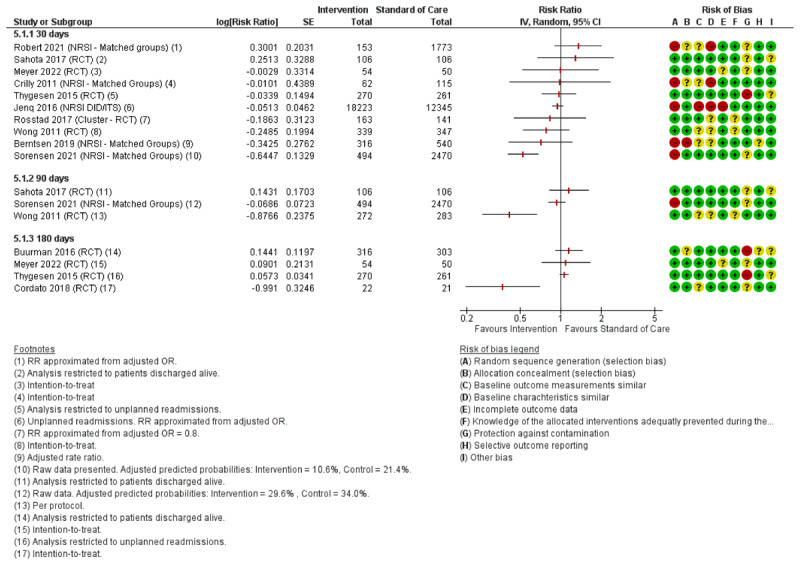
Forest plot of readmissions within 30–180 days. For the risk of bias assessment, red color indicates high risk of bias, yellow indicates unclear risk of bias and green indicates low risk of bias.

**Figure 3:**
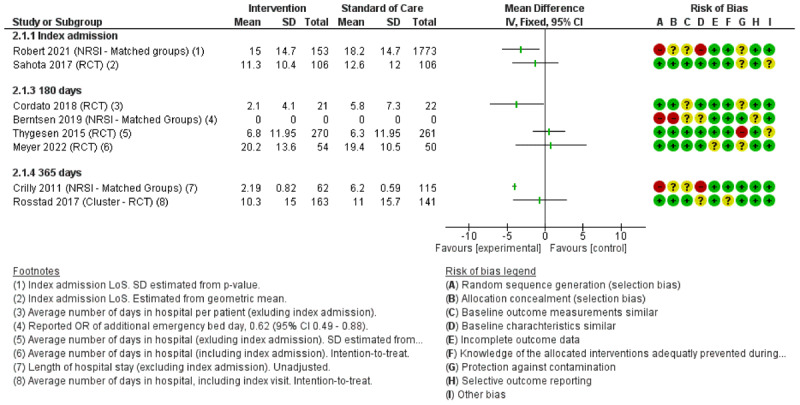
Forest plot with effect estimates for LoS at index admission and total number of days in hospital during 180–365 days.

**Figure 4:**
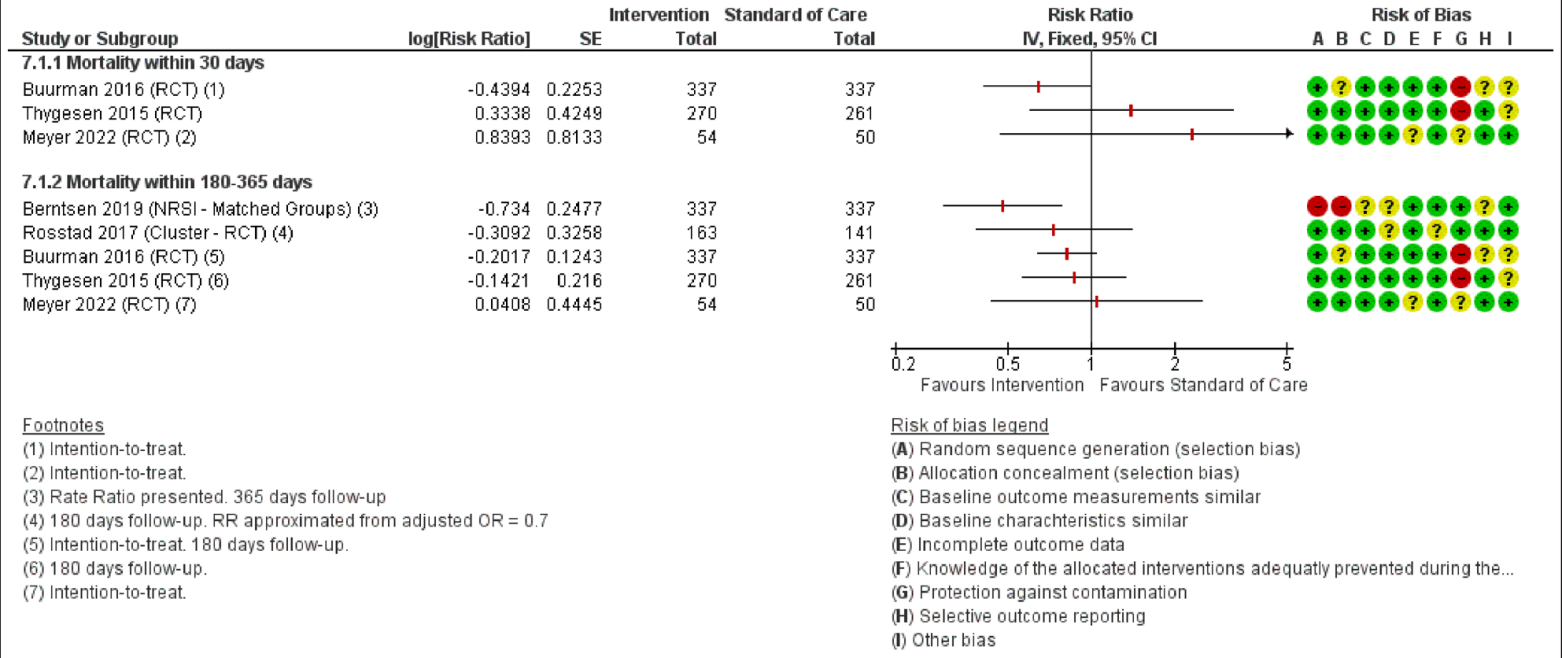
Forest plot of mortality within 30–365 days.

All twelve studies reported readmission rates with a follow-up period ranging from 30 to 180 days with eight studies reporting LoS (see Figures 2 and 3 respectively). Five studies reported mortality within 30–365 days, see Figure 4. Tables with QoL, ADL and other additional outcomes are presented in supplementary material A (*Supplementary tables 2 and 3)*.

#### Readmissions

For 30-day readmissions, seven out of ten studies reported decreasing trends of readmissions but only one study demonstrated statistically significant results [72]. Two studies reported trends of increased readmissions after 30 days (not statistically significant) [69,75]. For 90–180 day readmissions, three out of six studies reported decreased readmissions, with two studies reporting statistically significant results [67,70]. Four studies reported trends for increased readmissions (not statistically significant) [61,66,69,71].

#### Length of stay (LoS)

Three RCTs reported non-statistically significant decreases with mean difference (MD) point estimates ranging from a decrease of –0.7 to –3.7 days (not statistically significant) [67,68,69]. Two RCTs reported non-statistically significant increases [61,71]. The three NRSIs all reported decreases in length of stay, with two studies reporting statistically significant results [60,74,75].

#### Mortality

For mortality within 30 days, two out of three studies reported non-significant increases in mortality [61,71]. For mortality within 180–365 days, three RCTs reported non-significant decreases (RR ranging from 0.83 to 0.87) [61,66,68]. The NRSI reported a statistically significant decrease in mortality within 180 days (RR = 0.48) [60].

#### QoL and ADL

Four studies, all RCTs, reported QoL for follow-up periods ranging from 30 to 365 days [68,70,71,76]. Three studies reported increased QoL and one study reported no change. Four studies, all RCTs, reported ADL for follow-up periods ranging from 30 to 365 days. All studies reported increases in ADL point estimates, however none were statistically significant [66,68,69,70]. A summary of QoL and ADL effects is presented in the supplementary material A (*Supplementary table 2*).

#### Other outcomes

Six out of twelve studies reported outcomes related to emergency department (ED) utilization with three studies reporting a decrease in ED-visits (point estimates) [60,67,72]. The other three studies reported either a statistically non-significant increase in ED-visits or statistically significant increase in ED length of stay [61,74,75]. Four studies reported on the discharge destination post-discharge [61,66,68,71], with two studies reporting an increase (point estimates) in admissions to nursing homes [61,66]. One C-RCT reported a decrease in discharge to nursing homes within 365 days of discharge, however the effect was not statistically significant [68]. All four studies that reported outpatient visits reported increases in outpatient visits within 30 to 365 days [60,61,68,75]. Two studies reported statistically significant effects for follow-up periods ranging from 180 to 365 days [60,68]. A summary of effects on additional outcomes is presented in the supplementary material A (*Supplementary table 3*).

## Discussion

This systematic review is unique in that it focuses on a specific process, hospital discharge, within a particular context, where distinct and autonomous health and social care organizations collaborate to improve care for older patients.

In brief, the results illustrate that interventions mainly relied on needs assessments, dedicated care coordinators and multi-professional follow-up, which aligns with previous literature [42]. For the primary outcomes, the included studies reported both shorter LoS and decreased readmissions, demonstrating the potential effect of IOC on health care utilization. Mortality showed different patterns depending on the length of follow-up with increased mortality within 30 days (not statistically significant) and statistically significant decreases in mortality for longer follow-up periods. The studies evaluating effects on patient reported outcomes reported small improvements with no indications of worsening of QoL or ADL. In summary, these findings are largely consistent with previous studies, demonstrating the potential of coordinated care in decreasing health-care utilization while maintaining the patients well-being [67,70,72]. Despite the efforts in this study to specify a specific type of integrated care, the included studies were still highly heterogeneous which prevented us from fully answering the second research question.

Furthermore, the findings also reflect the inconsistent results found in other reviews of coordinated care reporting desired, undesired and non-effects [5,6,46,51]. Interestingly, statistically significant effects on LoS and readmissions were only reported from non-European countries, such as Australia and the US [67,70,72,74,75].

One possible explanation is that the studies from non-European countries more commonly reported the use of program theory, formal agreements and workflow improvements, indicating a higher awareness of interactions between intervention and the surrounding health-and social care system which has been reported as important in implementation of interventions of high complexity [77]. However, two European studies also reported such activities and although decreased readmissions, LoS and mortality was observed, the results were not statistically significant [60,68]. However, these results should be interpreted with caution as the non-European studies were the only studies that specifically targeted NH-residents [67,74] and utilizing pharmacists, which the literature suggests to be one of the most effective methods of decreasing readmissions in older adults. Furthermore, the studies conducted outside of Europe more commonly utilized non-randomized study designs, possibly overestimating the effects of these interventions [72,74,75].

An additional interesting aspect of the studies that reported non-clinical components is the stronger emphasis on both pre- and post-discharge plans and reconciliation meetings in comparison to other studies [60,68,70,72]. These results align with the importance of timing service provision, as missed or delayed care may be a sufficient cause of disproportionate changes in health status and function for this group of patients.

Finally, three studies reported increased readmissions (not statistically significant) [66,69,75], with two reporting simultaneous decreases in LoS [69,75]. As short LoS is a known risk factor for readmissions, these observations suggests that the post-discharge coordination was not successful in these interventions [69,75]. Alternatively, increases in readmissions may be due to an increased identification of unmet need following more intense follow-up post discharge.

Taken together, the findings in this systematic review suggest that non-clinical intervention components in conjunction with careful care planning during and after hospitalization may be important conditions for effective IOC. Further research is needed to establish the possible mediating role of non-clinical intervention components on the effectiveness of IOC.

### Strengths and limitations

The main strength of this review lies in its incorporation of contextual factors, in addition to the traditional PICO framework, which expands our understanding of how interventions operate in real world settings for older patients with complex needs. Previous systematic reviews have often overlooked the critical role of context, particularly for complex interventions that must interact with the surrounding health- and social care systems. Without considering these contextual elements, interventions may prove impractical or unfeasible to implement.

A related strength of the review is the inclusion of studies reporting both health care utilization and health related outcomes, generating a broad picture of the effects of IOC in comparison to reviews focusing on single outcomes as well being in better alignment with the goals of coordinated care [40,47]. Furthermore, this systematic review has the advantage of including articles with relatively strong study designs with comparatively low risk of bias in comparison to studies included in other reviews [6]. Indeed, the Risk of Bias assessments in this review indicates that the overall risk of bias in the included studies is rather low given the complexity of the interventions.

In order to avoid evidence selection bias it important that systematic reviews include all relevant studies [78]. Although several steps were taken to minimize selection bias in this review, e.g. searches in several databases informed by a trained information specialist and screening of relevant systematic reviews, the possibility of selection bias cannot be excluded. First of all, the search strategy may have missed relevant search terms as there is no consensus regarding definitions and technical terms in the field of integrated and coordinated care [79]. Secondly, the inclusion criteria regarding study design may have excluded relevant IOC-interventions, as these are sometimes evaluated using less reliable study designs. Furthermore, some studies that could be relevant for older patients may have been excluded because of the age criteria (i.e. patients older than 65 years of age).

Third, the authors consisted of a team with different backgrounds (physicians, health economists and health services researchers) with different views on what constitutes coordinated care. As such, the screening process and inclusion of full text articles required continuous discussions regarding which papers to include which is reflected in the Cohen’s kappa statistics. The main reasons for the relatively low agreement between authors were due to poor descriptions of context and uncertainty about the active participation of both in- and outpatient service providers. Although several sources were consulted (e.g. study protocols, process evaluations and corresponding authors) relevant articles may have been excluded [55].

Another concern in systematic reviews is related to publication bias where the tendency of not publishing negative or non-findings causes systematic reviews to overestimate the effects of interventions [80]. However, this does not seem to be a concern in this systematic review as unfavorable and non-significant effects were more common than beneficial effects.

Despite this, there are still some concerns regarding the included studies that could the interpretation of the results. For instance, even though several studies reported implementation activities [66,68,70,73,74,75], few studies described intervention fidelity. As such, it is not known whether the lack of desired effects in some studies were due to failures of the interventions or failures of their implementation [80,81]. Furthermore, some studies reported LoS as average number of days in hospital, not specifying if the analysis was made based on days in hospital per hospital visit or per patient, changes in LoS may therefore be indicative of changes in readmissions rather than length of hospital stay [61,67,68,71].

## Conclusion and policy implication

There are high expectations on IOC to manage the care needs of aging populations. From a policy perspective, the findings of this systematic review suggest that IOC indeed has the potential to decrease readmissions and LoS for older patients during hospital-to-home transitions. However, as several studies in this systematic review reported unexpected effects of IOC (e.g. increased short-term mortality) further research is needed in order to determine the most effective set of components for IOC.

## Involvement of people with lived experience

The authors have professional experience of configuring health care services for older patients with complex needs (RS, RSK) and evaluating coordinated care initiatives in the Swedish health care system (IF, UW). People with lived experience of being patients or users of integrated care services were not involved in this study.

## Additional Files

The additional files for this article can be found as follows:

10.5334/ijic.9018.s1 Supplementary Material A. Additional tables and figures.

10.5334/ijic.9018.s2 Supplementary Material B. Interactive version of table 3.
