# Supplementary material for: Inter-Organizational Coordination to Improve Patient Outcomes in Multimorbid Older Patients Following Hospital Discharge – a Systematic Review
Source: Int J Integr Care. 2025 May 12;25(2):12. doi: 10.5334/ijic.9018 (PMC12082463; doi:10.5334/ijic.9018)
Supplement: Supplementary Material A. — Additional tables and figures. [file ijic-25-2-9018-s1.pdf]

Supplementary material A

Modifications to protocol

The review aim was changed from investigating of *“collaboration between health- and social care providers”* to *“inter-organizational coordination between in-and outpatient care providers”* (including both health- and non-health care providers). The reasoning behind this change was that the services provided by social care workers are often targeted at vulnerable populations other than older patients and not relevant for this review focusing on older patients with multimorbidity (e.g. homeless individuals and individuals with substance use disorders). Furthermore, *collaboration* was changed to *coordination* as this term more accurately describes the type of processes used between independent service providers.

Furthermore, the stated aims in the protocol were not attainable given the small sample and heterogeneity of included studies. As such, the analytic second objective, stated in the protocol as *“What characterizes collaborative interventions that consistently show beneficial effects?”* was changed to a more modestly stated descriptive objective.

Example of search strategy

Supplementary Table 1. Search strategy in PubMed

Search number	Search terms	Number of records
1	aged[MeSH Terms] OR aged, 80 and over [MeSH] OR comorbidity[MeSH Terms] OR aged[Title/Abstract] OR elderly[Title/Abstract] OR frail elderly[Title/Abstract] OR oldest old [Title/Abstract] OR comorbid*[Title/Abstract] OR multimorbid*[Title/Abstract]	3,779,632
2	collaborat*[Title/Abstract] OR coordinat*[Title/Abstract] OR co-ordinat*[Title/Abstract] OR shared[Title/Abstract] OR integrat*[Title/Abstract] OR cooperat*[Title/Abstract] OR co-operat*[Title/Abstract] OR inter-organization*[Title/Abstract] OR inter organization*[Title/Abstract] OR inter-sectoral[Title/Abstract] OR inter-organisation*[Title/Abstract] OR inter organisation*[Title/Abstract] OR intersectoral[Title/Abstract] OR interinstitution*[Title/Abstract] OR inter institution*[Title/Abstract]	1,203,113
3	care[Title/Abstract] OR healthcare[Title/Abstract] OR health care[Title/Abstract] OR intervention*[Title/Abstract] OR service*[Title/Abstract] OR model*[Title/Abstract] OR effort*[Title/Abstract] OR manage*[Title/Abstract] OR approach*[Title/Abstract] OR practice*[Title/Abstract]	7,846,520
4	program evaluation[MeSH Terms] OR interprofessional relations[MeSH Terms] OR program evaluation*[Title/Abstract] OR programme evaluation*[Title/Abstract] OR intersectoral collaboration*[Title/Abstract] OR inter-sectoral collaboration*[Title/Abstract] OR interprofessional relation*[Title/Abstract]	150,371
5	2 AND 3 OR 4	730,596
6	patient care[MeSH Terms] OR nursing care[MeSH Terms] OR patient centered care[MeSH Terms] OR patient care[Title/Abstract] OR nursing care[Title/Abstract] OR patient centered care[Title/Abstract] OR proactive care [Title/Abstract] OR delivery of health care[MeSH Terms] OR delivery of health care[Title/Abstract] OR quality of health care[MeSH Terms] OR quality of health care[Title/Abstract] OR care coordination[Title/Abstract] OR care pathway*[Title/Abstract] OR complex intervention*[Title/Abstract] OR health service research[Title/Abstract] OR integrated care[Title/Abstract] OR health care utilization[Title/Abstract] OR person centred care[Title/Abstract	8,025,011
7	community health services[MeSH Terms] OR home care services, hospital-based[MeSH Terms] OR nursing homes[MeSH Terms] OR home care services[MeSH Terms]OR health services for the aged[MeSH Terms] OR primary health care[MeSH Terms] OR transitional care[MeSH Terms] OR community health service*[Title/Abstract] OR hospital-based home care services[Title/Abstract] OR nursing home*[Title/Abstract] OR municipality care[Title/Abstract] OR community care[Title/Abstract] OR caring home*[Title/Abstract] OR social care[Title/Abstract] OR social service*[Title/Abstract] OR home care service*[Title/Abstract] OR health services for the aged[Title/Abstract] OR transitional care[Title/Abstract] OR primary health care[Title/Abstract] OR community health nursing[Title/Abstract]	536,135

8	length of stay[MeSH Terms] OR patient readmission[MeSH Terms] OR mortality[MeSH Terms] OR length of stay[Title/Abstract] OR patient readmission[Title/Abstract] OR rehospitalization[Title/Abstract] OR delayed transfer[Title/Abstract] OR delayed discharge[Title/Abstract] OR duration of stay[Title/Abstract] OR healthcare utilization[Title/Abstract] OR bed day*[Title/Abstract] OR patient discharge[Title/Abstract] OR mortality[Title/Abstract]	1,139,306
9	1 AND 5 AND 6 AND 7 AND 8	2,204
10	9 AND Filter: 2010-	1,424

Summary of excluded studies

After full text review, 240 articles were excluded. The main reason for exclusion was due to non-eligible interventions (n = 82). The majority of these interventions consisted of care coordinators (or case managers) employed at hospitals, with outpatient service providers not actively participating in the intervention (i.e. outpatient service providers were passive recipients of the interventions).

The second most common reason was due to ineligible study populations (n = 39). Typically, these studies were not restricted to older patients and included all patients above 18 years of age and/or other vulnerable populations with specific needs (e.g. treatment of substance use disorder). Furthermore, several studies (n=22) were excluded as the participating in – and outpatient service providers were merged into integrated care organizations, i.e. investigating intra-organizational rather than inter-organizational coordination. A large number of studies (n=46) were excluded for several of the abovementioned reasons.

Finally, some (n=22) studies were excluded due to ineligible study design, mainly due to weak quasi-experimental study designs (e.g. no control group and single center studies with DiD designs). Some studies were also excluded as they did not report readmissions or LoS or were economic evaluations with insufficient reporting of outcomes (n=29).

Risk of bias assessments

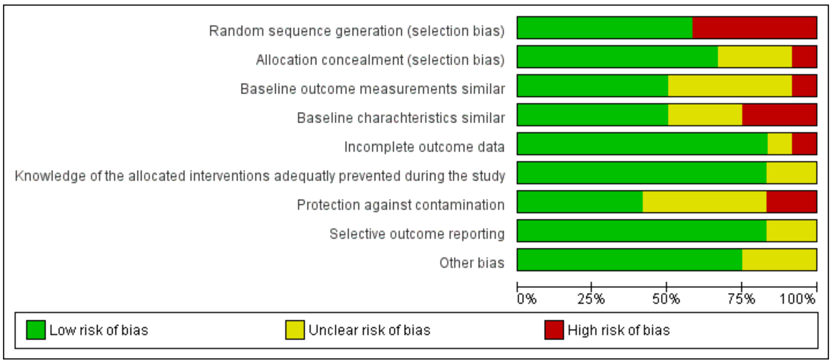

Supplementary figure 1. Review authors judgement about risk of bias items presented as percentages across all included studies.

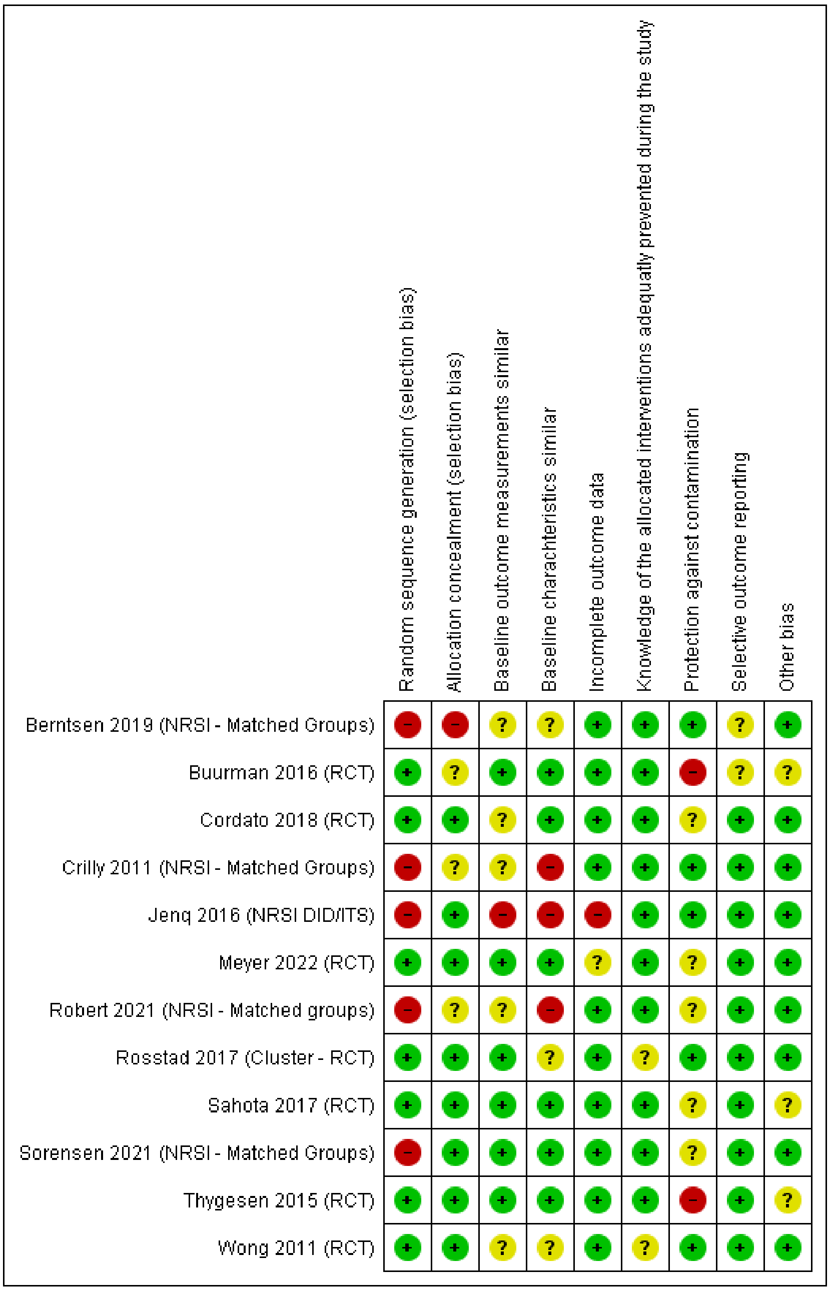

Supplementary figure 1. Review authors judgement about each risk of bias for each included study.

Red color indicates high risk of bias, yellow indicates unclear risk of bias and green indicates low risk of bias.

Additional outcomes

Supplementary table 2. Summary of findings for Quality of Life (QoL) and Activities of Daily Living (ADL)

Outcome	Study	Measurement	Follow-up (days)	Mean and SD at follow-up	Direction of effect
QoL					
	Rosstad 2017 (Cluster - RCT)	SF-36 (Mental Health)	365	Intervention = 46.7 (10.9), Control = 46.1 (12.5). MD = 1.1 (95% CI -2.6 - 4.8)	Increase
	Meyer 2022 (RCT)	EQ-5D-5L (index)	180	Intervention = 0.67 (0.44), Control = 0.53 (0.66). p-value = 0.284	Increase
	Sahota 2017 (RCT)	EQ-5D-3L	90	Not reported	No statistically significant difference
	Wong 2011 (RCT)	SF-36 (Mental health)	90	Intervention = 55.9 (10.7), Control = 54.5 (10.4). p-value =0.115	Increase
ADL)					
	Buurman 2016 (RCT)	Katz ADL	180	Intervention: 2.00 (95% CI 1.78 - 2.23), Control: 1.92 (95% CI 1.69 - 2.15). p-value = 0.32 (treatment x time interaction)	Increase
	Rosstad 2017 (Cluster - RCT)	NEADL (Mean, SD)	365	Intervention = 35.5 (17.1), Control = 32.1 (16.2). MD = 2.4 (95% CI −1.3 to 6.2)	Increase
	Sahota 2017 (RCT)	Barthel ADL (Mean, SD)	90	Intervention = 14.3 (5.5), Control = 12.6 (5.7). MD = 1.02 (95% CI -0.41 - 2.44)	Increase
	Wong 2011 (RCT)	Self-Efficacy (Mean, SD)	90	Intervention: 43.9 (11.9), Control = 41.4 (12.8). p-value=0.012	Increase

Supplementary table 3. Summary of findings for additional health care utilization outcomes

Outcome	Study	Measurement	Follow-up (days)	Effect	Direction of effect
**Discharge Destination**					
	Buurman 2016 (RCT)	Discharge to nursing home	180	Intervention = 16.1%, Standard of Care = 13.5%	Increase
	Rosstad 2017 (Cluster - RCT)	Nursing home admission (short and permanent stays) / patients	365	Adjusted Odds Ratio = 0.9 (95% CI, 0.7-1.3)	Decrease
	Thygesen 2015 (RCT)	Patients receiving nursing home services after index admissions	180	Intervention: 10%, Standard of care: 9%, p-value = 0.75	Increase
**Outpatient services**					
	Berntsen 2019 (NRSI - Matched Groups)	Planned Outpatient visits	180	Adjusted Rate Ratio = 2.26 (95% CI, 2.01 - 2.54)	Increase
	Robert 2021 (NRSI - Matched groups)	Follow-up with family physician	30	Adjusted Odds Ratio = 1.42 (95% CI, 0.90-2.24)	Increase
	Rosstad 2017 (Cluster - RCT)	GP encounters	365	Adjusted Odds Ratio = 1.4 (95% CI, 1.0 - 1.8)	Increase
	Thygesen 2015 (RCT)	Number of GP consultations	180	Intervention: 9.8, Standard of Care:	Increase

				9.2, p-Value = 0.18	
**Emergency Services**					
	Berntsen 2019 (NRSI - Matched Groups)	Count, Emergency outpatient visits (treat and release)	180	Adjusted Rate Ratio = 0.89 (95% CI, 0.67 - 1.18)	Decrease
	Cordato 2018 (RCT)	Episodes of care in ED	180	Intervention mean (SD): 0.6 (0.9), Standard of care mean (SD): 1.2 (1.3), p-value = 0,06	Decrease
	Crilly 2011 (NRSI - Matched Groups)	Length of Stay in ED (hours)	N/A	Adjusted Hazard Ratio = 1.65 (95% CI, 1.17 - 2.33)	Increase
	Robert 2021 (NRSI - Matched groups)	30-day ED visits	30	Adjusted Odds Ratio = 1.13 (95% CI 0.74 - 1.73)	Increase
	Sorensen 2021 (NRSI - Matched Groups)	30-day ED visit	30	Predicted probability (95% CI), Intervention: 10.4% (7.8 - 13.0), Standard of Care: 18.9% (17.4-20.5)	Decrease
	Thygesen 2015 (RCT)	Mean number of visits by emergency services doctor	180	Intervention: 1.0, Standard of Care: 0.7, p-value:0.19	Increase
